# The Preservation of All the Natural Teeth

**Published:** 1880-12

**Authors:** John T. Codman


### ARTICLE IV.

### *The Preservation of all the Natural Teeth.*

BY JOHN T. CODMAN, D. M. D.

Whatever I may present on this subject must be in the nature of hints rather than generous display of knowledge, and no attempt whatever will be made to cover *all* the ground that might be occupied to advantage. I promise to be brief and not trespass too much on the reader's time.

I take the ground that we as dentists should try to preserve all the natural teeth, and that this is our first duty.

Two professions at least should have something to say of the relations of the teeth to the system, to the health of the human body. These professions are represented by the man of anatomy, physiology and hygiene, and the dentist. The first should by the study of these kindred sciences be able to determine accurately the true physiological and physical conditions that must of necessity produce, develop and preserve teeth of fair structure. A study of these conditions will require a life time—must be continued from generation to generation until they are thoroughly understood and perfected. As to the second, the dentist, it is well that he should know all that the first knows or can know of these subjects, but this cannot be expected. The dentist's art absorbs his time and energies. The study of materials, the exercise of mechanical dexterity, and the daily toil for sustenance gives him enough to do as a dentist, without trespassing too far on ground that belongs more properly to another.

Now 1 know the reader will think it presumptous in me to propose to save all the natural teeth, and yet I think that to be our duty and no more than we should all strive accomplish. I grant that there are exceptions. As a sensible man I admit them, but these exceptions, to a student of dentistry, grow less and less as time and study go on. Dr. Flagg's "unbroken arches" will not in the future be such fabulous things to us or to a majority of the profession, who can hardily now beleve his statement a truthful one. I certainly can believe it not only possible, not only probable, but certain that with right materials, rightly used, thousands of teeth that to-day we are called upon to extract, may be preserved to a "good old age."

Then why are they lost? Simply because we are too ignorant to save them, or because we do not make the attempt. What is the condition of the teeth we are called upon to extract? In general, they are aching teeth, broken down teeth, or loose teeth. The greater portion are aching teeth, aching from inflammation of the pulp. This comes from various causes, such as disturbed constitutional conditions, stomachic, nervous, or otherwise. If these are reduced the pain disappears. If the pulp regains its health the tooth may be preserved. If the pulp is discased it may be destroyed, the root treated, and without a great deal of labor the tooth saved for years. Another portion are abscessed teeth. If the abscess is slight, proper treatment will reduce it. If greater, then with right treatment the abscess will become benign and the tooth and surrounding parts return to the normal condition, with the possible exception of a fistula that may heal after a while. Broken down teeth and roots deserve more thought and care than is usually given them. Why extract such when they are not troublesome ? This always produces shrinkage. When taken out, the arch that should be kept full is broken, and a thin look of the features with sunken cheeks is the result. Clean them, fill them with some of the plastic fillings, such as gutta percha, amalgam or oxychloride of zinc, if not with gold. Loose teeth may be preserved often times for years even when they seem hopeless; I know it for I have proved it. This is not theory ; it is what I have learned, what I try to do and accomplish in my practice.

If looseness is not occasioned by tartar, some other condition will be found that has raised the tooth or lowered it from its place in the socket. The strain produced by the closing of the jaws causes the foward and backward movement of this particular tooth and increases the tendency to elongate. The remedy is simple. File off the elongated tooth until free from its opponent. If necessary take some off its mate in the other jaw. In this way return it to restful conditions and let nature finish the work. She will accomplish more than many give her credit for.

But in this connection comes a matter of greater importance, and one on which I may find more disagreement than on any other point—the extraction of the sixth year molars to make room. I disagree with many on this point and adhere to the proposition, long since advanced, that the extraction of teeth from either jaw produces a narrowing of the arch. It cannot be otherwise. Then how do you gain room by so doing ? That we gain more room for teeth that still remain may be true, but I maintain that we have no right to extract for that purpose, except in very rare cases. "But," say some, "they are poor teeth and we cannot save them." If you cannot save them—if you cannot save teeth then do not call yourself a dentist. "But," say they "if I take them out I prevent the other teeth from crowding, and crowding is what makes teeth decay." Is it? Let us see. When I am shown an old person with sound teeth, or reasonably sound teeth, not close together nor crowded, then I may believe; but my experience is that no person of, say seventy years, can show a set of natural teeth unless such teeth are close together or crowded to lapping.

I could continue to write in this way, but do not wish to; but I ask why the lower animals have good teeth and the human animals poor teeth? And asking this question brings us at once to the "acid theory"—the theory stands better than any other. Sour mouths, sour stomachs, sour breaths, and I may add sour dispositions, are consistent with rotten teeth, slimy mouths *ad nauseum*, getting the better of tooth brushes, washes and tinctures every time.

Let us be radical as well as sensible and say what we ought to have said a thousand times: *The best diet of man is that which keeps his teeth clean and preserves them from decay.* As we are not physiologists, we do not know andh ave not studied the question of diet. We turn to those who have, to aid us, and demand of them to show us a diet that will meet all desired conditions. Let this be done and our work will stand, our fillings stay, and however crowded the teeth may be, such teeth will remain sound. I for one beg of any one to let me know and let the world know why I cannot have a diet that will keep my teeth as sound as those of the horse, as white as those of the dog, as clean as those of the cat. We as dentists will promise to double our zeal, to give our mechanical aid, our professional skill, as well as our hearts to our part of the problem if others will but do their duty by the part left to them.

In conclusion, I wish to say that sufficient attention has not been given the fact of the alteration of the shape of the face and the tones of the voice by the loss of the back teeth, and by the non-development of the dental arches. One of the great Italian writers on music says: "You cannot be a good singer unless you have all your teeth," or words to that effect. I wish this were inscribed in every household where the love of harmony exists. It is only by the development of all the teeth that the mouth is formed for harmony. Only by the development of the jaw, produced by the presence of wisdom teeth, is the uvula and the soft palate lifted out of the throat to its proper, harmonious place. Looking at the subject in this light, let us be more cautious how we extract teeth—more careful and persistent in our efforts to preserve them.—*Odontographic Journal.*

